# Regional environmental heterogeneity under contrasting anthropogenic pressures has differential effects on particle-attached than free-living bacteria communities in coral reef waters

**DOI:** 10.1128/spectrum.03078-25

**Published:** 2026-04-30

**Authors:** Dan He, Heng Wang, Lijuan Ren, Hao Luo, Jianglang Duan, Zhuo Chen, Qinglong L. Wu

**Affiliations:** 1 Research Center for Marine Biology and Ecology, Southern Marine Science and Engineering Guangdong Laboratory (Guangzhou) 638106 , Guangzhou, Guangdong Province, China; 2 Institute of Hydrological Biology, Jinan University 47885, Guangzhou, Guangdong Province, China; 3 Nanjing Institute of Geography and Limnology, Chinese Academy of Sciences 66289 , Nanjing, Jiangsu Province, China; China Agricultural University 34752, Beijing, China

**Keywords:** coral reef ecosystem, anthropogenic pressure, particle-attached bacteria, free-living bacteria, community network

## Abstract

Coral reef ecosystems are facing increasing environmental and anthropogenic pressures worldwide. Their health and prosperity depend, at least partly, on the surrounding water environment, where the free-living (FL) and particle-attached (PA) bacterial communities play essential yet distinct ecological roles. However, these communities’ distributions and variations across coral reef ecosystems with large environmental and anthropogenic gradients remain scarcely understood. In this study, we investigated FL and PA communities in coral reef waters from three regions of the South China Sea (SCS): Hainan, ZhongXisha, and Nansha. Among the various factors influencing bacterial communities, the region exhibited the greatest impact when compared to lifestyle (PA versus FL) and layer (surface versus bottom water). The Hainan waters exhibited higher contents of total organic carbon (TOC) and ammonia yet lower temperature, which likely contributes to the higher alpha diversity and the increase of Alphaproteobacteria and Bacteroidetes. While stochastic processes showed dominance for communities’ assembly, the relative importance of homogeneous selection increased particularly for the Hainan PA community. Network analyses showed that the Hainan PA community network was more complex and densely connected than other community networks. The anthropogenic (TOC and ammonia as proxies) and environmental contexts in the region of Hainan may elevate the robustness of the PA network but reduce that of the FL network. In conclusion, our results indicated that the regional environmental heterogeneity under contrasting anthropogenic pressures had differential effects on PA than FL bacteria communities in coral reef waters, which may provide clues for understanding ecosystem resilience and targeted management strategies for coral reef conservation.

**IMPORTANCE:**

Particle-attached (PA) and free-living (FL) represent two basic lifestyles for waterborne bacteria. While the knowledge about their differences in community diversity, assembly processes, and network structure is increasing, their variations and responsive patterns in coral reef waters in a large area with environmental heterogeneity and anthropogenic pressure differences remain scarcely understood. Here, we investigated their community dynamics in coral reef waters in the South China Sea. We found that the regional environmental heterogeneity had more prominent effects on PA than FL communities. In Hainan waters with lower temperature and higher contents of TOC and ammonia-N, the PA other than FL communities showed a more connected and robust network structure. The rich organics and specialized microniches within particles might help PA communities adapt and resist environmental perturbations under high anthropogenic pressure. Our results would help provide insights into understanding ecosystem resilience and inform targeted management strategies for coral reef conservation.

## INTRODUCTION

Coral reef ecosystems are characterized by high productivity and biodiversity, providing essential services such as fisheries, coastal protection, and tourism (1). The health and prosperity of coral reef ecosystems rely on their surrounding environment. Among them, the surrounding water bacteria play pivotal roles in driving biogeochemical cycling, including the decomposition of organic matter and the recycling of nutrients. These bacteria can also serve as the “seed bank” for coral-associated bacteria and may modulate the coral-microbe symbioses, thereby influencing the overall stability of reef ecosystems (2, 3).

The waterborne bacteria mainly live in two lifestyles: particle-attached (PA) bacteria and free-living (FL) bacteria. The PA bacteria colonize particulate organic matter and suspended aggregates, which were empirically found to be larger than 3 μm in size (4, 5). The particulate microenvironments create hotspots for microbial activity, high community diversity, and complex interactions (6). In contrast, FL communities, which inhabit the water column independently of particles, often exhibit lower diversity and activity in the decomposition of organic matter (7, 8). Previous studies have shown that these two kinds of bacteria were spatially separated and generally different in community composition and functions (9). For example, Gammaproteobacteria and Bacteroidetes were more abundant in PA communities, while FL communities were enriched with Alphaproteobacteria in the Northwest Mediterranean Sea (10). PA bacteria could harbor more genetic loci for the decomposition of complex polysaccharides (11). However, in the waters surrounding coral reefs, mucus and detritus released by corals and associated organisms can significantly influence both PA and FL microbial communities, potentially driving distinct patterns in their community composition and ecological dynamics compared to those observed in open pelagic seawater.

Anthropogenic influences on coral ecosystems, stemming from nutrient loading, agricultural runoff, overfishing, coastal urbanization, and plastic pollution, have been shown to modify environmental conditions significantly (12, 13). These stressors can have a profound impact on bacterial communities in coral reef water columns by altering their composition and assembly processes. For instance, shifts in water bacterial communities due to nutrient enrichment or the input of organic matter may trigger the proliferation of opportunistic taxa and dysbiosis, destabilizing microbial functions critical for coral health (14, 15). While knowledge about the impacts of anthropogenic pressures on coral reef ecosystems is accumulating, we still have limited understanding of how PA and FL bacterial communities in the surrounding waters may respond to these pressures. Their differential responses and the underlying mechanisms may provide clues for understanding ecosystem resilience and targeted management strategies for coral reef conservation.

The coral reefs in the South China Sea (SCS), encompassing regions from around Hainan Island to the Nansha Islands, provide an ideal natural laboratory to explore the distribution and dynamics of PA and FL bacterial communities subjected to different anthropogenic pressures. Hainan Island is densely populated and industrialized, exposing its nearby coral reefs to intense anthropogenic pressures, including urbanization, agricultural development, and tourism expansion (16). Conversely, the Zhongsha, Xisha, and Nansha Islands, located in remote, less-developed regions of the SCS, experience relatively pristine local conditions but remain susceptible to global-scale stressors, such as climate change, ocean warming, and plastic dispersal (17, 18). These contrasting conditions make these regions particularly valuable for comparative studies that examine the differential impacts of localized and global anthropogenic stressors on microbial communities in coral environments.

In the present study, we investigated the distributions of PA and FL bacterial communities in coral reef waters in the SCS. By leveraging the contrasting anthropogenic context of coral reefs, we seek to assess how environmental factors shape the surrounding water-bacterial communities at a large geographical scale. We hypothesized that (i) PA and FL bacterial communities showed disparate patterns in diversity, composition, and assembly processes; (ii) region (with contrasting anthropogenic pressures) may overrun the lifestyle and water layer in affecting communities’ variation; and (iii) PA communities might show higher adaptation and resistance than FL communities to anthropogenic pressures. This work will help fill knowledge gaps in coral-related microbial responses to human stressors and provide insights for coral reef management and conservation.

## MATERIALS AND METHODS

### Site description and sampling

**Fig 1:**
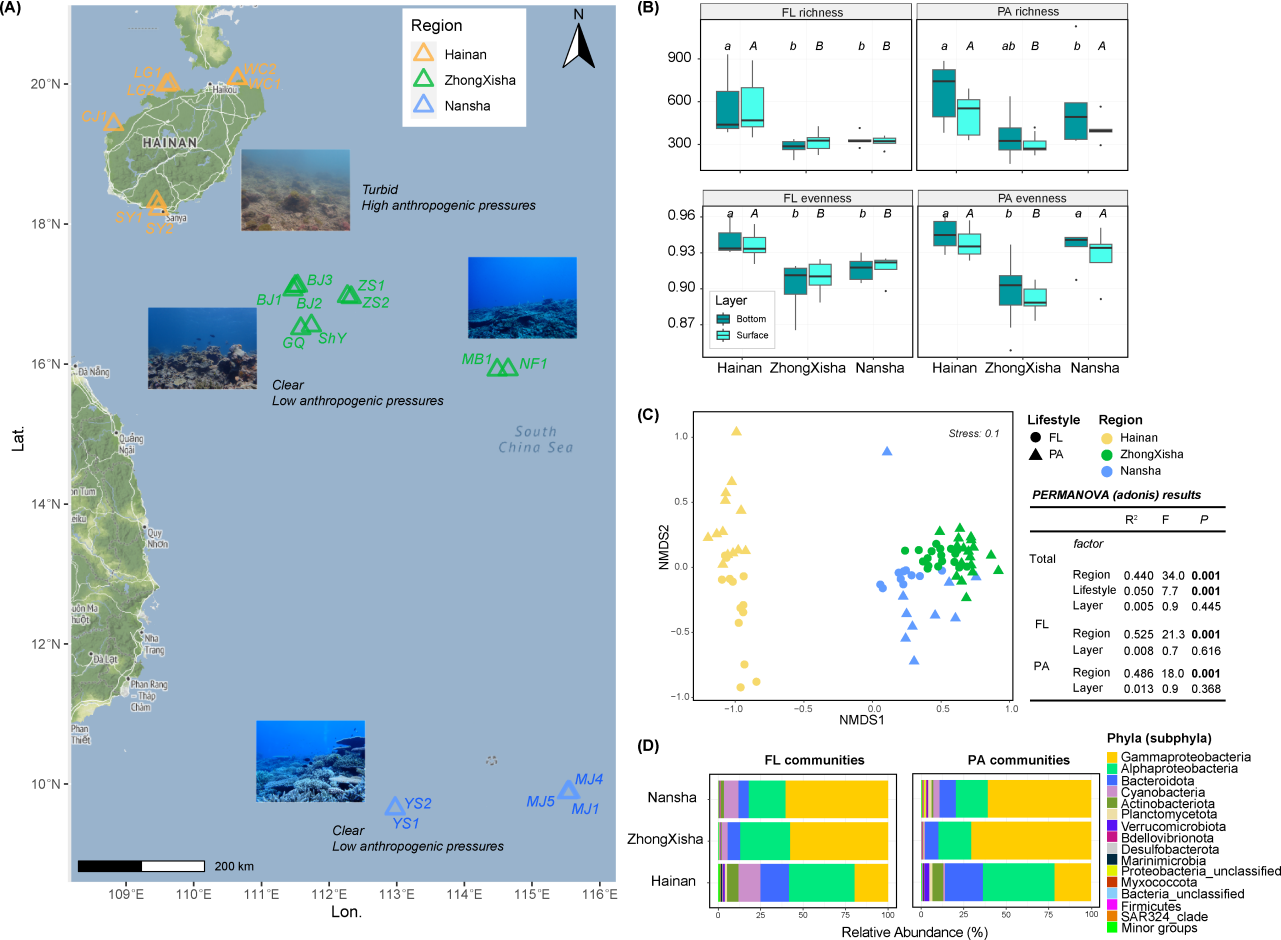
The sampling map and the diversity information about bacterial communities. (**A**) The map showing sampling sites and representative pictures of the coral waters. (**B**) The alpha diversity of free-living (FL) and particle-attached (PA) bacterial communities. Boxplots of richness and Pielou’s evenness are displayed for different regions, lifestyles, and water layers. Box boundaries = 25th (Q1) and 75th (Q3) percentiles; the black solid line inside denotes the median (50th percentile). Whiskers extend to the min/max values within Q1 − 1.5 × IQR and Q3 + 1.5 × IQR (IQR = Q3 − Q1). Orange solid dots outside whiskers are outliers (values beyond Q1 − 1.5 × IQR or Q3 + 1.5 × IQR). No common letters above the main boxes indicate no significant differences (perm *t*-test, FDR-adjusted *P* < 0.05) among different regions for the bottom and surface layer, respectively. (**C**) The NMDS plot illustrating the differentiation of bacterial communities across various lifestyles and regions. (**D**) The phyla (subphyla) compositions of different bacterial communities. All less abundant taxa (below the top 15) were put together to the “Minor groups” ranging from 0.005% to 2.58% in relative abundance in different samples.

From February 23 to May 9 in 2023, we collected water samples from the coral reefs surrounding Hainan Island and the small islands in the SCS. The representative coral reefs are located at seven sites in Hainan, two sites in the Zhongsha Islands, seven sites in the Xisha Islands, and five sites in the Nansha Islands (Fig. 1A). Considering the distances between different sites, we combined the Zhongsha and Xisha samples into one region group, referred to as ZhongXisha. At each site, nearly 5 L of seawater were collected from the surface (0.2–1.0 m depth) and the surrounding bottom waters, respectively. The depth of the sampling sites ranged from 2.01 m to 6.32 m in Hainan, from 4.50 m to 20.46 m in ZhongXisha, and from 9.49 m to 21.53 m in Nansha. The dominant coral species were *Porites*, *Euphyllia*, and *Acropora* (arranged by relative abundance) in Hainan; *Alcyonacea*, *Pavona*, and *Porites* in ZhongXisha; and *Acropora*, *Alcyonacea*, and *Porites* in Nansha. However, due to sampling limitations, the accurate identities of coral species in the water samples taken during SCUBA diving were not determined. The basic water properties were measured at each water depth using a YSI ProDSS water quality probe (YSI Incorporated, Yellow Springs, OH, USA).

### Samples processing

In the laboratory, nearly 500 mL of water was subsampled from each water sample. The subsamples were stored at −20°C and analyzed further for other physicochemical properties. The attached bacteria were collected by filtering (using a vacuum pump) 4.5 L through three 3 μm filters (PTFE, Millipore) and three 0.2 μm filters (GTTP, Isopore) sequentially. All the filters were stored at −80°C before further analyses.

### Physicochemical properties determination

In the laboratory, the contents of water PO_4_^3−^ were determined by ammonium molybdate spectrophotometry; the contents of water NO_3_^−^ and NH_4_^+^ were determined via continuous colorimetric flow analysis (Skalar SAN PLUS system, the Netherlands); the contents of water total organic carbon (TOC) were determined with a TOC analyzer (ET-1020A, Euro Tech).

### DNA extraction and sequencing

One of the triplicate filters was processed using the FastDNA SPIN Kit for Soil (MP Biomedicals). After extraction, the NanoDrop One (Thermo Scientific) was used to check the DNA quantity and purity. The highly purified DNA was then sequenced on the MiSeq platform in the Majorgene company (Guangzhou). The primers 515F (5′-GTGYCAGCMGCCGCGGTAA-3′) and 806R (5′-GGACTACNVGGGTWTCTAAT-3′) were used to sequence the 16S rRNA gene fragments of bacteria.

### Sequence processing

The paired sequences were merged using DADA2, implemented in the QIIME 2 (19). All the merged sequences were purified by setting the *Q* value threshold to 20 and the window size to 4. Then, the DADA2 generated amplicon sequence variant (ASV) tables with the default settings. The representative ASV sequences were mapped to the Silva v138 database (https://www.arb-silva.de/documentation/release-138/) for taxonomic assignment. All sequences and ASVs assigned to “chloroplast,” “mitochondria,” and “unknown” in taxonomy were discarded for further analysis.

### Bioinformatic analyses

The ASV tables and taxonomy files were imported into R (4.2.2) (https://www.r-project.org/). To further reduce the biases caused by ASVs with rare sequences, ASVs with fewer than 2 sequences were discarded before further analysis. The communities were then rarefied to contain the same number (20,813) of sequences for all samples. The ASV richness and Pielou’s evenness were calculated for each sample using the “vegan” package (version 2.6-10) (20). The Bray-Curtis dissimilarity was calculated based on the square root of the ASV abundances with the “vegdist” function in the “vegan” package. The DESeq (21) and Limma (22) methods were used to find the differentially abundant ASVs for different regions (Hainan versus the other regions). The ASVs with an absolute value of logFC (logarithm of the fold change) over 5 and an adjusted *P*-value (Benjamini-Hochberg) less than 0.001, calculated by both two methods, were determined as the differentially abundant ASVs.

To understand the assembly processes, Raup-Crick dissimilarity and null model-based phylogenetic analyses were performed for FL and PA samples, respectively. These analyses were conducted within the group of each region for FL and PA communities, respectively. The relative importance of the five assembly processes (homogeneous selection, heterogeneous selection, dispersal limitation, homogenizing dispersal, and drift) was then calculated (23). These analyses were conducted using the “iCAMP” (24) package in R.

The community network was constructed based on the ASV table, which included the top 2,000 bacterial ASVs for the PA (41 samples in total) and FL communities (42 samples in total), respectively. The Spearman correlation coefficients within PA or FL communities were calculated for each ASV pair. The significant and real network links were estimated sequentially with the “SparCC” method (25) and the deconvolution method (26). The generated correlation pairs were then input into R, and the network was generated using the “igraph” package (27). Sub-networks were extracted from the meta-network by attaining the ASVs in the specified groups. The region-specific sub-network files (nodes and edges worksheets) were then imported into Gephi (v0.10) for visualization. The network-level and node-level traits were then calculated by different functions in the “igraph” package. Their description can be found in Table S2. We calculated two robustness indices according to the method depicted by Yuan et al. (28). The robustness_random was measured as the proportion of nodes remaining after 50% of the nodes randomly removed from each network, and the robustness_target was measured as the proportion of nodes remaining after 15 nodes (randomly chosen from the top 30 nodes based on their degrees) removed from the original network.

### Statistical analyses

The significance of differences between groups in environmental properties, community alpha diversity, and network node traits was tested using permutation-based analysis of variance (perm-ANOVA) (conducted by the “aovp” function in R) or *t*-tests (perm *t*-test). *P*-values for multiple comparisons were adjusted for false discovery rate (FDR) correction. The grouping effects of different factors on community compositions were tested using a permutation-based multivariate analysis of variance (PERMANOVA, conducted by the “adonis” function in R). The variance partition analyses (VPA) of community compositions were done with the “varpart” function in R. The important factors or environmental variables for alpha (represented by the “Shannon” diversity) and beta diversity (represented by the principal coordinates of community dissimilarity) were identified using the random forest method. The effect of geographical distance was reflected by the principal coordinates of neighborhood matrix (PCNM). All statistical analyses were performed using R (version 3.3.2) with the following packages: “stats,” “vegan,” “lmPerm” (v2.1.0) (29), “coin” (30), “agricolae” (v1.3-7) (31), and “randomForest” (32).

## RESULTS

### Seawater environment properties

**Fig 2:**
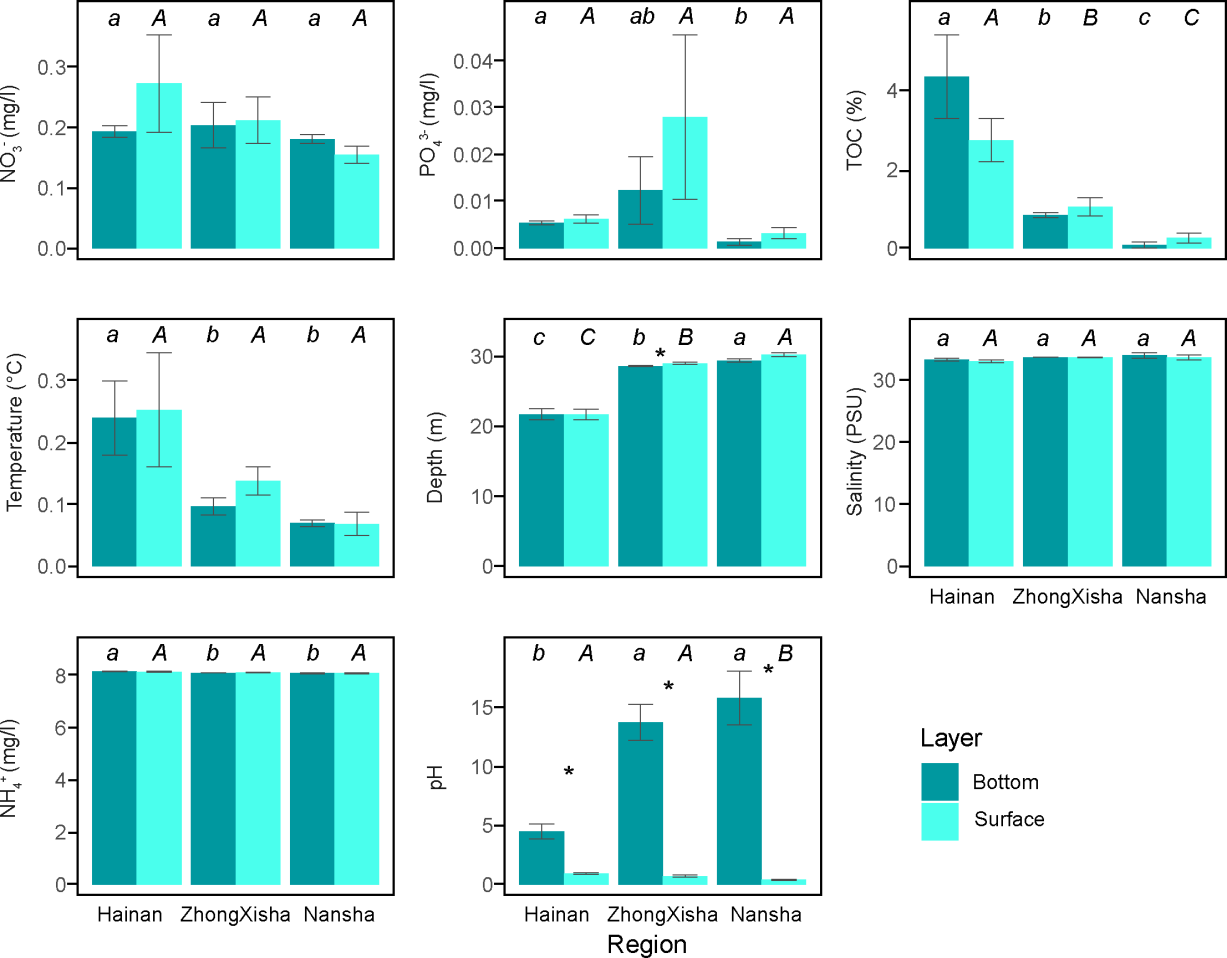
The environmental properties of the coral reef waters measured in this study. No common letters above the boxes indicate no significant differences among different regions for the bottom and surface layers, respectively (perm *t*-test, FDR-adjusted *P* < 0.05). The “*” above bars for each region denotes significant differences between the bottom water and surface water (perm *t*-test, *P* < 0.05). TOC, total organic carbon; Temp, temperature; Dep, depth.

The studied Hainan seawater showed significantly higher values of TOC, NH_4_^+^, and pH, while exhibiting significantly lower values of temperature compared to ZhongXisha and Nansha (mainly in comparing bottom waters) (Fig. 2). In contrast, the latter two regions showed no significant differences for most of the examined properties, except that Nansha had a higher temperature than ZhongXisha (perm *t*-test, FDR-adjusted *P* < 0.05) (Table S3). Generally, no properties showed significant changes between the bottom and surface waters except depth itself.

### PA and FL bacterial community diversity

The average values of ASV richness ranged from 281.1 to 669.7. The three-way perm-ANOVA analyses showed that region had a significant (*P* < 0.05) effect, while lifestyle (FL versus PA) had a marginally significant (*P* < 0.1) effect, and layer had no significant effect on community richness (Fig. 1B) (Table S4). The region had a significant effect (*P* < 0.05), while lifestyle and layer had no significant effect on community evenness. There were no significant differences between different lifestyles or different layers for both richness and evenness. Generally, Hainan showed significantly higher values for both richness and evenness than the other two regions, although no significant differences were detected between Hainan and Nansha (perm *t*-test, FDR-adjusted *P* < 0.05) (Table S5). The Venn plots showed that the overlap of PA and FL communities was higher in Hainan (16.5%) than that in ZhongXisha (14.9%) and Nansha (14.7%).

As the NMDS plot showed (Fig. 1C), region had the major (PERMANOVA, *R*^2^ = 0.38, *P* < 0.001), and lifestyle had the secondary (PERMANOVA, *R*^2^ = 0.05, *P* < 0.001), while layer had no significant effects (PERMANOVA, *R*^2^ = 0.005, *P* > 0.05) on the compositional changes of bacterial communities in different samples. For different lifestyles, FL communities showed a little higher compositional changes (*R*^2^ = 0.53) across different regions than did the PA communities (*R*^2^ = 0.49).

### The phyla abundances and differentially abundant ASVs

At the phylum (subphylum) level, there were large differences in the relative abundances between Hainan and the other two regions, while the latter two showed generally similar patterns (Fig. 1D). The relative abundances of Gammaproteobacteria were significantly lower (mean ± SD, 20.7 ± 5.7%) in Hainan than in ZhongXisha (64.2 ± 5.7%) and Nansha (60.6 ± 9.6%), while Alphaproteobacteria, Bacteroidota, and Actinobacteriota were significantly more abundant in Hainan than in ZhongXisha and Nansha (perm *t*-test, FDR-adjusted *P* < 0.05) (Table S6). Lifestyle played a secondary role in affecting the relative abundances of bacterial phyla. For example, Cyanobacteria and Marinimicrobia_(SAR406_clade) were significantly more abundant in FL than in PA communities, while Planctomycetota, Verrucomicrobiota, and Firmicutes were significantly more abundant in PA than in FL communities in at least two regions (perm *t*-test, FDR-adjusted *P* < 0.05) (Table S6) (Fig. 1D).

**Fig 3:**
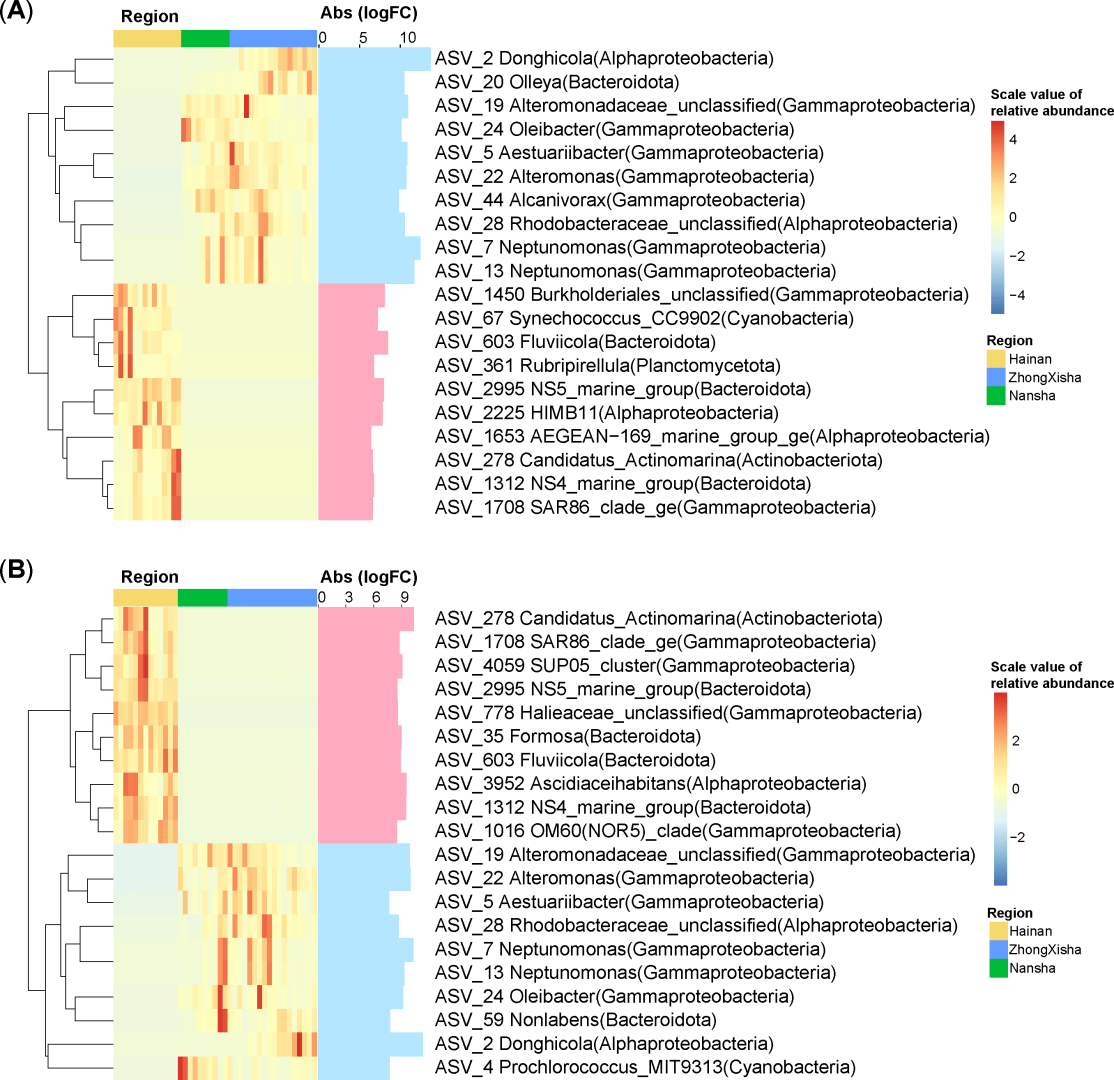
The differential/discriminant analyses between Hainan and the other two regions. The analyses were done for (**A**) free-living and (**B**) particle-attached communities, respectively, using the methods “DESeq” and “Limma.” The differentially abundant amplicon sequence variants (ASVs) with the top 10 absolute values of logFC (logarithm of fold change) were shown in the heatmap. The scaled relative abundances of these ASVs were clustered using the method “hclust” in the left panel of the heatmap. The light blue and light red colors mean negative and positive logFC values, respectively, when comparing Hainan with the other two regions. Abs(logFC), the absolute value of the logFC.

The two methods (DESeq and Limma) identified a total of 56 differentially abundant (log fold change > 5) FL ASVs and 62 PA ASVs between Hainan and the other regions. There were five ASVs (ASV_603, ASV_2995, ASV_278, ASV_1312, ASV_1708) that were differentially abundant for both PA and FL communities in the Hainan region. They were affiliated with the taxa *Fluviicola*, NS5_marine_group, NS4_marine_group, SAR86_clade, and Candidatus_Actinomarina. Eight of the top 10 (fold change) ASVs were differentially abundant for both PA and FL communities in ZhongXisha and Nansha. They were affiliated in the taxa such as *Neptunomonas* (ASV_7, ASV_13), *Alteromonas* (ASV_22), *Aestuariibacter* (ASV_5), *Oleibacter* (ASV_24), *Donghicola* (ASV_2), and Rhodobacteraceae (ASV_28) (Fig. 3). The other top differentially abundant ASVs were affiliated with taxa including HIMB11 (Alphaproteobacteria, ASV_2225) and Synechococcus_CC9902 (Cyanobacteria, ASV_67) for Hainan FL communities; *Alcanivorax* (Gammaproteobacteria, ASV_44) for FL communities in ZhongXisha and Nansha; *Ascidiaceihabitans* (Alphaproteobacteria, ASV_3952), *Formosa* (Bacteroidota, ASV_35), and SUP05_cluster (Gammaproteobacteria, ASV_4059) for Hainan PA communities; and Prochlorococcus_MIT9313 (Cyanobacteria, ASV_4) for PA communities in ZhongXisha and Nansha.

### Important factors in driving community changes

**Fig 4:**
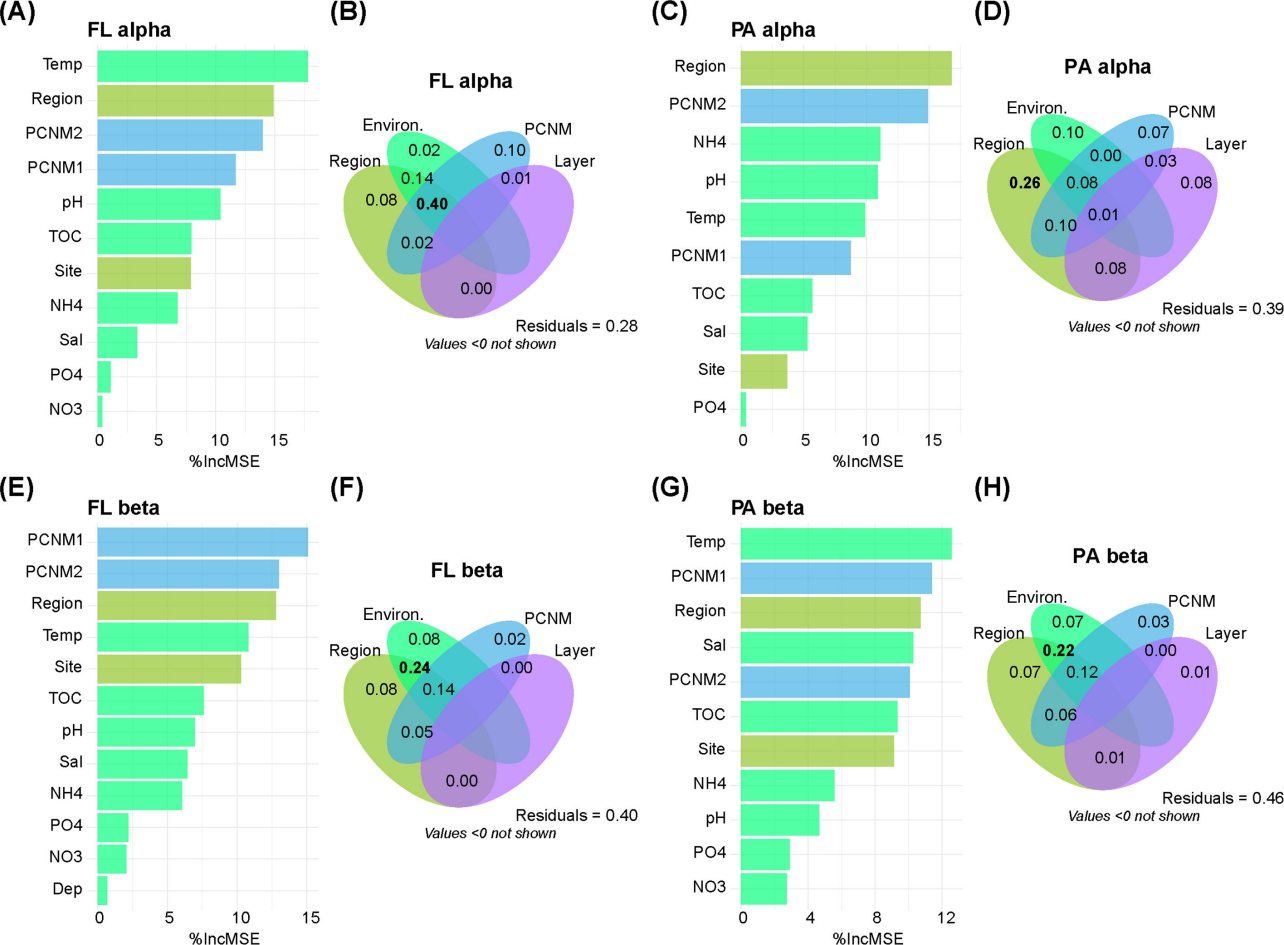
The random forest and variation partition analyses for bacterial diversity changes. All factors and environmental properties were involved in the random forest analyses for the alpha diversity of free-living (**A**) and particle-attached (**C**) communities, and the beta diversity of free-living (**E**) and particle-attached (**G**) communities, respectively. The IncMSE in the random forest plot means the increase of mean squared error when the variable is randomly permuted, indicating its importance to model prediction accuracy. The factors region and layer, the total group of environmental properties, and the first two principal coordinates of neighborhood matrix (PCNM) for geographical distance were involved in the variation partition analyses (VPA). VPA was done for the alpha diversity of free-living (**B**) and particle-attached (**D**) communities and the beta diversity of free-living (**F**) and particle-attached (**H**) communities, respectively. The largest proportion of explained variance was shown in bold. FL, free-living; PA, particle-attached.

The random forest analyses showed that region and geographical distance (represented by PCNM1 and PCNM2) played primary roles in affecting alpha and beta diversity across all communities. For FL communities, environmental properties, such as temperature, pH, and TOC, were important for both alpha and beta diversity (Fig. 4A and E). For PA communities, environmental properties, such as NH_4_^+^, pH, and temperature, were important for alpha diversity, while temperature, salinity, and TOC were important for beta diversity (Fig. 4C and G).

The variation partition analyses were also done for both alpha and beta diversity. For alpha diversity, the combination of region, environmental properties, and geographical distance (PCNM) (40%) explained the largest part of the variation for FL communities, while the pure effect of region (26%) explained the largest part of variation for PA communities (Fig. 4B and D). For beta diversity, the combination of region and environmental properties explained the largest part of community variation, followed by the combination of region, environmental properties, and geographical distance (PCNM) for both FL and PA communities. The pure effects were similar between environmental properties and region, which were larger than those of geographical distance. The pure effects of layer were trivial (Fig. 4F and H).

To further tease apart the effects of anthropogenic-driven environment and other environmental variables, we conducted partial mantel analyses to link PA and FL community changes. The results showed that, for both PA and FL communities, temperature had the strongest correlation with community changes, while the anthropogenic proxy variables (such as NH_4_^+^ and TOC) showed secondary importance in correlating with community changes (Table S7).

Water TOC contents correlated positively with the relative abundances of Alphaproteobacteria, Bacteroidota, and Verrucomicrobiota, and negatively with those of Gammaproteobacteria and unclassified Proteobacteria for both PA and FL communities. Temperature and salinity showed converse correlations to TOC with these phyla (Fig. S2). pH showed a greater number of significant correlations with FL communities (such as Actinobacteriota, Firmicutes, and Planctomycetota) than PA communities. NH_4_^+^ showed positive correlations with some FL phyla (such as Bacteroidota and Acidobacteriota) but negative correlations with some PA phyla (such as Cyanobacteria and Bdellovibrionota). PO_4_^3^⁻ showed a negative correlation with Cyanobacteria in PA communities.

### Community assembly processes

The phylogenetic null-model based analysis showed that, generally, the ecological drift had higher importance than all other processes in shaping bacterial community assembly (Fig. S3). However, there were also differences between different regions or lifestyles. For example, the relative importance of homogeneous selection (18.2%) increased exclusively in the assembly of PA communities from Hainan. Homogenizing dispersal showed higher importance for FL communities than for PA communities. On the contrary, dispersal limitation showed higher importance for PA communities than for FL communities.

### Community network structures and keystone species

**Fig 5:**
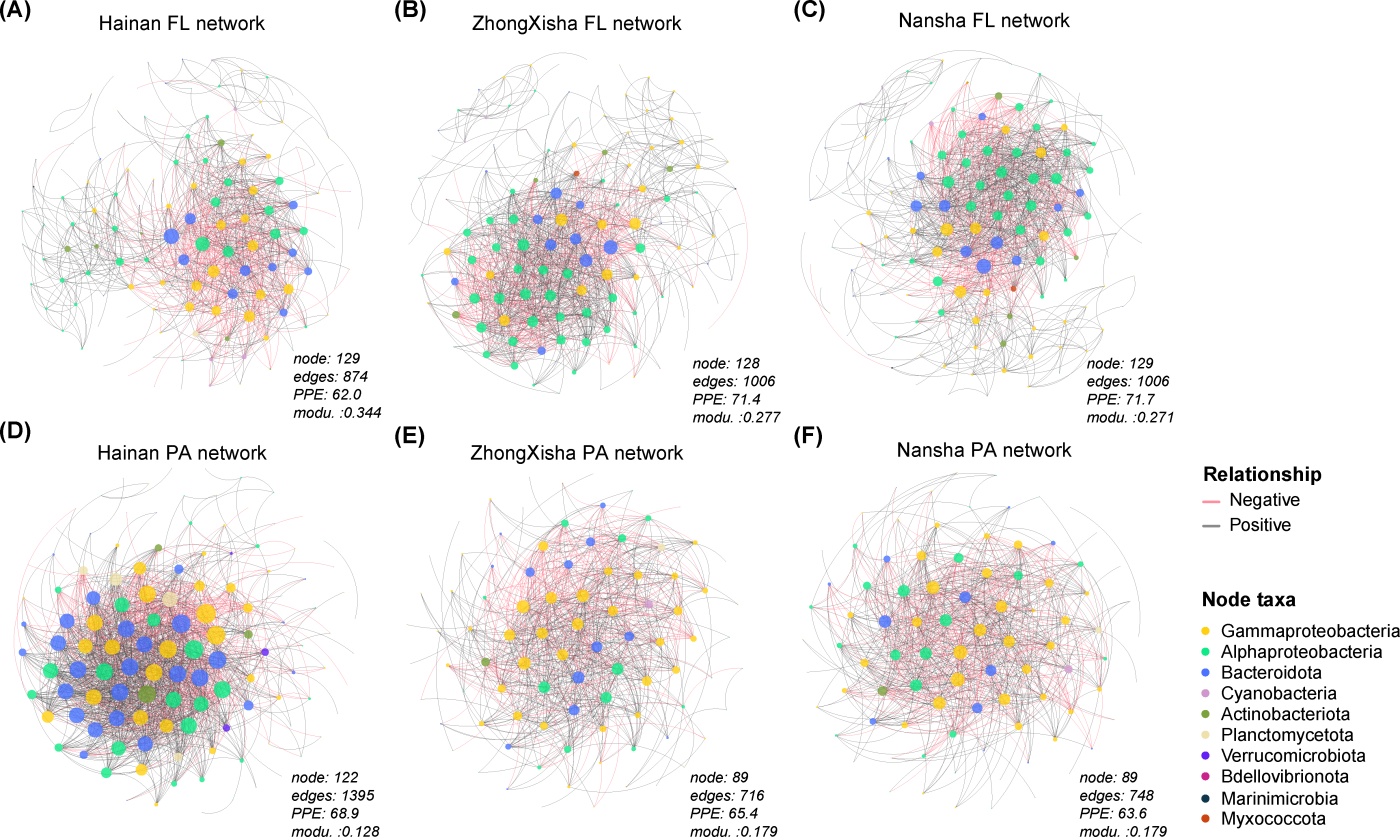
The network topological structures and the *Zi-Pi* plots showing keystone species for communities from different regions and lifestyles. (**A–C**) The network topology for FL communities from (**A**) Hainan, (**B**) ZhongXisha, and (**C**) Nansha. (**D–F**) The network topology for PA communities from (**D**) Hainan, (**E**) ZhongXisha, and (**F**) Nansha. The node size was proportional to its degree, and the node color was based on its taxa group at the phylum (subphylum) level. PPE, percent positive edge; Modu., modularity; FL, free-living; PA, particle-attached.

The ASV co-occurrence networks were constructed for the PA and FL bacterial communities, respectively, and subsequently, a total of six region-specific sub-networks were generated with the “induced_subgraph” function in R. The sample numbers ranged from 10 to 18 for these sub-networks (Table 1). The network topological structures were similar between ZhongXisha and Nansha for both PA and FL community networks. They exhibited disparate patterns compared to those from Hainan (Fig. 5). At the network level, for FL networks, Hainan showed a less connected (lower degree) and more modular structure (higher modularity) than the other regions. Conversely, for PA networks, Hainan showed a more connected and less modular structure than the other regions (Table 1, Fig. 5A through F). At the node level, all FL networks consistently showed lower values of closeness and eigen-centrality than those of PA networks for all regions; Hainan showed significantly higher degrees and Knn (average nearest neighbor degree) values than the other regions for PA networks (permT-test, all cases, FDR-adjusted *P* < 0.05) (Table S8).

**TABLE 1:** the basic network-level traits for the six region-lifestyle specific networks^*a*^

	Hainan_FL_net	ZhongXisha_FL_net	Nansha_FL_net	Hainan_PA_net	ZhongXisha_PA_net	Nansha_PA_net
Sample number	14	18	10	13	18	10
Node number	129	128	129	122	89	89
Edge number	874	1,006	1,006	1,395	716	748
Mean degree	13.55	15.72	15.60	22.87	16.09	16.81
Mean distance	2.78	2.85	2.84	2.31	2.22	2.17
Edge density	0.11	0.12	0.12	0.19	0.18	0.19
Modularity	0.34	0.28	0.27	0.13	0.18	0.18
robustness_rd (mean ± SD)	0.669 ± 0.016	0.723 ± 0.017	0.747 ± 0.015	0.601 ± 0.027	0.554 ± 0.020	0.578 ± 0.020
robustness_tgt (mean ± SD)	0.354 ± 0.025	0.382 ± 0.023	0.395 ± 0.023	0.336 ± 0.025	0.331 ± 0.029	0.333 ± 0.030

^*a*^ The descriptions about these traits are introduced in the (Table S2) robustness_rd, the robustness calculated by randomly removing the nodes from the original network. robustness_tgt, the robustness calculated by targeted removing the nodes (based on their degree values) from the original network.

The *Zi-Pi* plots (Fig. S5) indicated that there were more keystone species in PA networks than in FL networks, with the number peaking in the Hainan PA network. Almost all keystone species fell into the category of “connectors” (Zi < 2.5 and Pi > 0.62). The abundant and common keystone species primarily originated from Gammaproteobacteria (such as *Alteromonas*, *Marinomonas,* and *Oceanimonas*) and Bacteroidota (including Flavobacteriaceae) across all PA networks. For the Hainan PA network, Planctomycetes (such as *Rubripirellula* and *Blastopirellula*) and Alphaproteobacteria (such as *Ascidiaceihabitans*) were also abundant among keystone species. The keystone species affiliations were very similar for the ZhongXisha and Nansha FL networks, which mainly came from Alphaproteobacteria (such as SAR116_clade) and Myxococcota (Blfdi19). While for the Hainan FL network, keystone species were primarily from the Bacteroidota (Flavobacteriaceae) and Gammaproteobacteria (such as Pseudomonadaceae and Halieaceae).

**Fig 6:**
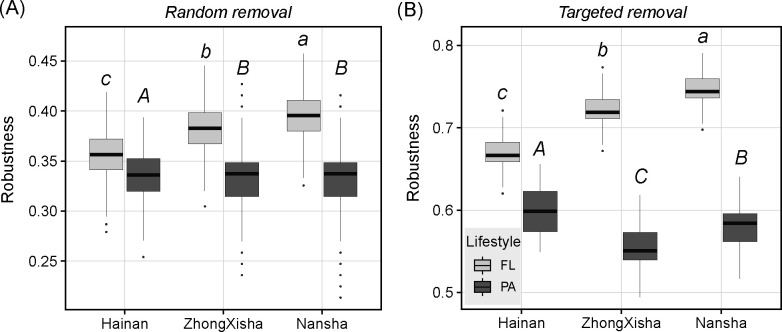
Robustness is calculated by (**A**) random removal and (**B**) targeted removal of the nodes in different networks. Box boundaries = 25th (Q1) and 75th (Q3) percentiles; the black solid line inside denotes the median (50th percentile). Whiskers extend to the min/max values within Q1 − 1.5 × IQR and Q3 + 1.5 × IQR (IQR = Q3 − Q1). Orange solid dots outside whiskers are outliers (values beyond Q1 − 1.5 × IQR or Q3 + 1.5 × IQR). No common letters above the bars denote no significant differences (perm *t*-test, FDR-adjusted *P* < 0.05) of the mean values between different regions. There were significant differences between different lifestyles (perm *t*-test, *P* < 0.05) for all regions.

For the two robustness indices, the PA networks exhibited significantly lower values than the FL networks for all regions (perm *t*-test, FDR-adjusted *P* < 0.05) (Table S9). By comparing different regions, the PA network showed the highest robustness in Hainan than in other regions. On the contrary, the FL network showed the lowest robustness in Hainan than in other regions (perm *t*-test, FDR-adjusted *P* < 0.05) (Fig. 6, Table 1).

The correlation analyses revealed that, except for the environmental properties NO_3_⁻, PO_4_^3^⁻, and depth, all other properties exhibited significant correlations with both the keystone species’ compositions and the network-level traits (Mantel test, all cases, *P* < 0.05). Temperature and TOC were the two variables that exhibited stronger correlations with network traits and keystone species distribution (Fig. S6).

## DISCUSSION

This study explored the community distribution patterns of PA and FL bacteria in coral reef waters subjected to regional environmental heterogeneity under different anthropogenic pressures in the SCS. We found that the two kinds of bacteria exhibited disparate scenarios in terms of diversity, assembly processes, and co-occurrence network structures, which could be attributed to their differential lifestyles and the influence of anthropogenic pressures. The results would help deepen our understanding of seawater bacterial communities in coral reef ecosystems in a changing world.

### Factors driving bacterial community changes at a large geographical scale

The PA and FL represent two different lifestyles for water bacteria. It had been reported that PA communities were more diverse than FL communities, and they showed apparent deviations in taxonomic compositions (11, 33). In one previous study, the effect of lifestyle was more prominent than space (approximately 20 km) in affecting marine bacterial communities (34). However, in this study, when the space expanded to a large geographical scale (over 1,000 km) with clear anthropogenic pressure differences, the region played the most dominant role, while lifestyle played a secondary role in driving the changes of bacterial communities. The alpha diversity was significantly higher in Hainan than in other regions but changed very little between different lifestyles in each region (Fig. 1B). The secondary role of lifestyle was further witnessed in the NMDS plot, where the bacterial samples firstly grouped by regions and then by lifestyles, which were supported by the PERMANOVA analysis (Fig. 1C). PA and FL communities could exchange some members as the particle aggregates assemble and disassemble (35). The exchange extent of their communities (ASVs overlap) might be enhanced in Hainan, where the total carbon contents were significantly higher than the other two regions (Fig. 2). A similar trend of convergence between FL and PA communities was also observed in the lake, where the eutrophic region had higher concentrations of nutrients and organic particles (8).

The region’s effects in this study may stem from differential anthropogenic pressures, environmental variables (such as temperature), and other unmeasured regional factors in Hainan and the other two regions. The VPA analyses showed that the combination of region and environmental properties explained the largest variation in community compositional changes, indicating that regional environmental heterogeneity exerted a great effect on community beta diversity. By contrast, the pure effects of region (spatial separation) and environment (teasing out the spatial difference) were similar, and both were much less than their combined effect (Fig. 4F and H). Among the water environmental properties, temperature, TOC, salinity, and pH were the top important properties for community compositional changes (Fig. 4E and G). The partial mantel analysis confirmed that temperature was the greatest variable affecting PA and FL communities, while the anthropogenic “proxy” variables, such as TOC and NH_4_^+^, only shed secondary effects. Temperature represents a basic environmental property that regulates bacterial metabolism and community compositions in coral waters (36). This dominant role of temperature indicates the fundamental regulating effects of environmental heterogeneity at a large geographical scale on seawater bacterial communities. The water TOC content was typically higher in Hainan than in other regions (Fig. 2), owing to the terrestrial inputs of organic particles through runoffs and rivers to the sea (37). The higher TOC and NH_4_^+^ contents could help sustain higher bacterial alpha diversity in Hainan (Fig. 1B and 2), probably due to sufficient substrate availability of carbon and nitrogen (38, 39). Nutrients’ effect on alpha diversity might be enhanced in PA than FL communities (Fig. 4A and C).

The water layer (surface and bottom) had minor effects on bacterial diversity and compositions (Fig. 1B and C). The high dispersal rate of seawater due to horizontal and vertical flows (40) may downsize the effects of layer. However, in the specific region of Hainan, the effects of layering increased. For example, the TOC contents were higher in bottom water than in surface water in Hainan, which may help drive the increase of alpha diversity in bottom waters for PA communities other than FL communities (Fig. 1B and 2). This result also suggested that PA alpha diversity may be more sensitive to anthropogenically driven environmental changes (such as the increasing of organic matter).

The phyla that were reported to be highly abundant in the PA communities in previous studies, such as Gammaproteobacteria and Bacteroidetes (11, 41), were not found to be highly abundant in this study (Fig. 1D). As discussed above, the lifestyle is subordinate to the region in driving phyla composition differentiation. Alphaproteobacteria and Bacteroidetes were more abundant in Hainan for both PA and FL communities. The two taxa are highly correlated with TOC contents (Fig. S2). Some Alphaproteobacteria were shown to better utilize low-molecular-weight DOC (such as amino acids and dimethylsulfoniopropionate [DMSP]) than Betaproteobacteria and Gammaproteobacteria (42, 43), which may reveal some clues for the organic matter constituents in Hainan coral waters (44). Five and eight of the top 10 differential ASVs were shared between PA and FL communities for Hainan and the other two regions, respectively, which also evidenced the dominant effect of region over lifestyle (Fig. 3). The *Formosa* and the NS4 and NS5 marine groups had unique genes for the degradation of polysaccharides (45); their differential abundance may reflect the utilization of glycan targets corresponding to those structurally identified in POM in Hainan waters. *Fluviicola* was considered to be characteristic of freshwater (46), and their differential abundance might result from the input of freshwater from the Hainan Island. The *Ascidiaceihabitans* and Halieaceae discriminated in Hainan PA communities were reported to be differentially abundant in biofilm or bioflocs (47, 48), which might also show a preference to niches in organic particles. The *Alteromonas* was reported to be enriched with coral mucus addition (49) and may represent typical bacteria in coral reef waters.

### Different assembly processes of the PA and FL communities

Compared to a study on soil bacterial communities at a large spatial scale (50), we also observed the dominance of stochastic processes in driving seawater bacterial communities (Fig. S3). As expected, homogenizing dispersal was more important for the FL community assembly than for the PA community, because FL bacteria, being smaller in size and mass, might migrate farther through active movement and/or passive transportation (51). The importance of homogeneous selection was over 14% for all samples, which might be associated with the selection from the coral reef environment. The mucus and detritus excreted by corals and other organisms possibly shed selection effects on bacteria differently from those in open water (52). The relative importance of selection increased apparently in driving the assembly of Hainan PA communities, suggesting that anthropogenic pressure (such as organic input) might add to the effect of selection on PA, rather than FL bacteria, given the higher concentration of organics in particles compared to the surrounding water (53). The top five bins (cumulatively over 55% contribution) contributing to homogeneous selection mainly came from the genera SAR11 Clade_Ia (Alphaproteobacteria), Candidatus_Actinomarina (Actinobacteriota), uncultured Cryomorphaceae (Bacteroidota), and Erythrobacter (Alphaproteobacteria) (Table S10). They all showed positive correlations with water TOC content and pH and a negative correlation with water temperature. These genera were typical marine taxa, often exhibiting adaptive specialization in organic resource utilization and marine biofilm development (54–57).

### PA communities rather than FL communities showed more complex and robust network structures under high anthropogenic pressures.

Generally, PA networks showed a higher degree but lower modularity than FL networks (Fig. 5; Fig. S4), consistent with previous studies (58), which suggest that particles represent a niche where bacteria may have more interactions in a relatively defined space than in open water. This difference was amplified in Hainan networks (Fig. 5). The high amount of organic particles may induce cooperation between different members of PA communities to maximize the utilization of the organic matter (59). At the node level, PA networks exhibited higher closeness and eigen_centrality than FL networks across different regions, indicating that members of PA communities had closer relationships and more efficient potential interactions with one another than those in FL communities. The degree and KNN were significantly higher in Hainan PA networks compared to the other networks, further suggesting that the potential interactions were more complex and intense in Hainan PA communities.

For a community network, the metric robustness indicates the stability of the community structure to external perturbations (60). The PA networks consistently showed lower robustness than the FL networks for all regions (Fig. 6), which contrasted with those results for PA and FL networks in previous lake studies (61, 62). Some reasons might explain this inconsistency. First, the alpha diversity of PA and FL communities was not significantly different in our study (Fig. 1B); thus, the stability originating from high alpha diversity and its buffering effect against the loss of individual species was not guaranteed for PA communities, unlike what has been observed in previous lake studies. Second, the PA networks were less modular than FL networks for all regions (Fig. 5, Table 1). External perturbations could more readily transmit along the whole network when it is highly centralized. However, robustness is a complex index that is associated with the whole structure of the network. In addition to the modularity, other network traits, such as degree, network density, and interaction type/strength, may also contribute to the network robustness (63, 64). Notably, we found that the robustness was especially elevated in the PA network and reduced in the FL network in Hainan compared with other regions. At similar levels of modularity, network density and interaction type may contribute to the differences in network robustness between regions for PA and FL communities, respectively (Fig. 5). Generally, the higher the density of a network, the more stable it becomes when its nodes are randomly removed or attacked. The differential trends in robustness for PA and FL networks in Hainan suggest that the high anthropogenic pressures could have contrasting effects on PA and FL community stability. The high amount of organics might promote the relationship between FL community members to shift from symbiosis to competition (65). Species loss due to environmental filtering and competition might result in a less stable network structure in Hainan FL communities. On the contrary, PA bacteria generally showed higher adaptation and growth rates than FL bacteria under high organic carbon conditions in water (66, 67). The sufficient C and N substrate and specialized microniches may endow the PA bacterial community with greater stability against external perturbations (68).

The *Zi-Pi* plots showed that there were more keystone species in PA networks, especially in the PA Hainan network (Fig. S5), indicating that within the defined particle space, more hub species were capable of mediating interactions within communities. The number of hub species might increase under high anthropogenic pressures and special environments in the Hainan region(Fig. S5D). Most of the keystone species of PA networks were affiliated in the Gammaproteobacteria genera, such as *Alteromonas*, *Marinomonas*, and *Oceanimonas,* and the Bacteroidota family Flavobacteriaceae. The taxonomic affiliations might provide some clues for community organization and their functional adaptation to the external environment. The *Alteromonas* are typical bacteria capable of cell-cell aggregation and biofilm formation (69). Bacteria affiliated with Oceanimonas and Marinomonas were reported to possess *dddD* genes to produce DMSP. These bacteria may play essential roles in the sulfur cycling within the particle niche. The Flavobacteriaceae are renowned for their ability to degrade high-molecular-weight complex organic matter, which may provide other members with a readily usable carbon source (70). The *Rubripirellula* have been reported to be enriched in plastic particles in the Baltic Sea (71), and their specificity for the keystone role in the Hainan PA network implied plastic pollution in the studied Hainan waters (72, 73). Although to a lesser extent, the anthropogenic pressures and special environmental conditions in the Hainan region also affect the distribution of keystone species in FL networks. The SAR116 and SAR86 lineages are typical free-living marine bacteria; some members of these lineages have been reported to have the ability to degrade DMSP, which may mediate sulfur cycling in the FL communities (74).

### Conclusion

The bacterial communities in reef waters play essential roles in ecosystem function and the health of corals. In this study, we investigated the FL and PA bacterial communities in the surrounding waters on a large spatial scale in the SCS. Among the driving factors, the region played a dominating role in affecting the bacterial community in terms of diversity, composition, and network traits. Lifestyle (FL or PA) played a secondary role, while layer (bottom or surface water) had minimal effects. The region’s effects might have stemmed from the differential anthropogenic pressures between Hainan and the other regions. The higher organic matter content in Hainan waters correlated with higher alpha diversity and the relative abundances of Alphabacteria and Bacteroidetes in both FL and PA communities, with the latter showing a more prominent effect. While stochastic processes dominated the assembly processes of all communities, the importance of homogeneous selection increased particularly for Hainan PA communities, indicating that higher anthropogenic pressures (such as high organic input) might have greater selection effects for PA communities than for FL communities. What is more, the high anthropogenic pressure and environment properties in Hainan showed differential effects on the robustness of the network for PA than FL communities, elevating that of the PA network but reducing that of the FL network compared with other regions. The distinct response patterns to regional environmental heterogeneity under different anthropogenic pressures between FL and PA fractions highlight their adaptation to distinct ecological niches and may provide clues for understanding ecosystem resilience and inform targeted management strategies for coral reef conservation.

## Data Availability

The original bacterial 16S rRNA gene sequences were deposited in the sequence archive (https://submit.ncbi.nlm.nih.gov/subs/sra/) under the BioProject number PRJNA1148802. The datasets and codes for this study can be accessed at https://github.com/Dan01He/PA-and-FL-bacteria-communities-in-coral-reef-waters/.

## SUPPLEMENTAL MATERIAL

Supplemental material spectrum.03078-25-s0001.docx

ASM does not own the copyrights to Supplemental Material that may be linked to, or accessed through, an article. The authors have granted ASM a non-exclusive, world-wide license to publish the Supplemental Material files. Please contact the corresponding author directly for reuse.
