# Supplementary material for: Regional environmental heterogeneity under contrasting anthropogenic pressures has differential effects on particle-attached than free-living bacteria communities in coral reef waters
Source: Microbiol Spectr. 2026 Apr 30;14(6):e03078-25. doi: 10.1128/spectrum.03078-25 (PMC13228049; doi:10.1128/spectrum.03078-25)
Supplement: Supplemental material [file spectrum.03078-25-s0001.docx]

**Supplementary Materials**

for the manuscript entitled

“Regional environmental heterogeneity under contrasting anthropogenic pressures has differential effects on particle-attached than free-living bacteria communities in coral reef waters”

**Authors:** Dan He ^1^, Heng Wang ^1^, Lijuan Ren ^2,*^ , Hao Luo ^1^, Jianglang Duan ^1^, Zhuo Chen ^1^, Qinglong L. Wu ^1,3,*^

^1^ Research Center for Marine Biology and Ecology, Southern Marine Science and Engineering Guangdong Laboratory (Guangzhou), Guangzhou, Guangdong Province, China, 511458

^2^ Institute of Hydrological Biology, Jinan University, Guangzhou, Guangdong Province, China, 510632

^3^ Nanjing Institute of Geography and Limnology, Chinese Academy of Sciences, Nanjing, Jiangsu Province, China, 211135

^*^ Corresponding author.

*E-mail address*: lijuanren@jnu.edu.cn (Lijuan Ren); wu_qinglong@gmlab.ac.cn (Qinglong L. Wu)

Short summary: This file contains 10 supplementary tables (Table S1-S10) and 7 supplementary figures (Figure S1-S7).

**Table S1.** The sampling sites and stations in this study. The top 5 abundant coral taxa in each station (if available) were shown.

| **Station** | **Site** | **Region** | **Lat.** | **Lon.** | **Main coral taxa** |
| --- | --- | --- | --- | --- | --- |
| SY1 | Sanya | Hainan | 18.3197222 | 109.4505556 | *Porites*, *Euphyllia*, *Turbinaria*, *Tubastrea*, *Pocillopora* |
| SY2 | Sanya | Hainan | 18.2150000 | 109.4780556 | *Porites*, *Euphyllia*, *Acropora*, *Psammocora*, *Pocillopora* |
| CJ1 | Changjiang | Hainan | 19.4230555 | 108.8155556 | not available |
| LG1 | Lingao | Hainan | 20.0019444 | 109.6477778 | *Euphyllia*, *Porites*, *Acanthastrea*, *Bernardpora* |
| LG2 | Lingao | Hainan | 20.0013888 | 109.6561111 | *Porites*, *Euphyllia*, *Turbinaria*, *Goniopora* |
| WC1 | Wenchang | Hainan | 20.0791666 | 110.6444444 | *Acropora*, *Porites*, *Turbinaria*, *Favites*, *Goniastrea* |
| WC2 | Wenchang | Hainan | 20.0775000 | 110.6408333 | *Euphyllia*, *Pocillopora*, *Acropora*, *Echinopora* |
| NF1 | Nanfei | ZhongXisha | 15.9213500 | 114.6456333 | *Euphyllia*, *Acropora*, *Psammocora*, *Pavona*, *Rugosa* |
| MB1 | Manbu | ZhongXisha | 15.9166000 | 114.4829500 | *Pocillopora*, *Acropora*, *Porites*, *Alcyonacea*, *Pavona* |
| Shy | Shiyu | ZhongXisha | 16.5486833 | 111.7384500 | *Alcyonacea*, *Porites*, *Pavona*, *Heliopora* |
| GQ | Ganquan | ZhongXisha | 16.5087611 | 111.5811639 | *Alcyonacea*, *Pocillopora*, *Porites*, *Gorgonacea*, *Echinopora* |
| ZS | Zhaoshu | ZhongXisha | 16.9678833 | 112.2709278 | *Acropora*, *Alcyonacea*, *Porites*, *Pavona*, *Favites* |
| XS4 | Zhaoshu | ZhongXisha | 16.9520833 | 112.3264722 | *Alcyonacea*, *Acropora*, *Pavona*, *Montipora*, *Psammocora* |
| BJ1 | Beijiao | ZhongXisha | 17.0624338 | 111.4668618 | *Alcyonacea*, *Heliopora*, *Rugosa*, *Dipsastraea* |
| BJ2 | Beijiao | ZhongXisha | 17.1164225 | 111.5334214 | *Pavona* |
| BJ3 | Beijiao | ZhongXisha | 17.1108080 | 111.4998780 | *Heliopora*, *Alcyonacea*, *Pavona*, *Psammocora*, *Pocillopora* |
| YS1 | Yongshu | Nansha | 9.6644361 | 113.0147167 | *Acropora*, *Dipsastraea*, *Porites*, *Millepora* |
| YS2 | Yongshu | Nansha | 9.6577956 | 112.9762171 | *Pocillopora*, *Alcyonacea*, *Acropora*, *Millepora*, *Porites* |
| YS3 | Yongshu | Nansha | 9.6449500 | 112.9774056 | *Acropora*, *Alcyonacea*, *Pocillopora*, *Porites*, *Dipsastraea*, |
| MJ1 | Meiji | Nansha | 9.8908333 | 115.5353333 | not available |
| MJ4 | Meiji | Nansha | 9.8836527 | 115.5603944 | *Acropora*, *Porites*, *Alcyonacea*, *Isopora*, *Seriatopora* |
| MJ5 | Meiji | Nansha | 9.8772750 | 115.5214111 | *Acropora*, *Alcyonacea*, *Rugosa*, *Montipora*, *Pachyseris* |

**Table S2.** The descriptions about the network traits used in this study.

|  | **Term** | **Description** |
| --- | --- | --- |
| node-level traits |  |  |
|  | Degree | Number of edges incident to the vertex. |
|  | Closeness | Reciprocal of the average shortest path distance from a node to all other nodes in the network, measuring how close a node is to the center of the network. |
|  | Betweenness | The frequency with which a node appears on the shortest paths between all pairs of nodes, measuring the importance of a node as a "bridge". |
|  | Eigenvector Centrality | Measures node importance based on the importance of its neighbors, where connections to important nodes contribute more to the score. |
|  | Average Nearest Neighbor Degree (Knn) | The average degree of all neighbors of a node, reflecting the tendency of a node to connect with highly connected or poorly connected nodes. |
|  | Percent postive edges (PPE) | The proportion (percent) of positive edges in all edges linking to the node, reflecting strength of positive correlations with its neighbors |
| network-level traits |  |  |
|  | mean_distance | Calculated as the average of the shortest paths between all pairs of nodes in the network. |
|  | mean_degree | Calculated as the sum of all node degrees divided by the number of nodes. |
|  | centr_betw | Calculated as the normalized sum of differences between the maximum betweenness and each node's betweenness. |
|  | centr_clo | Calculated as the normalized sum of differences between the maximum closeness and each node's closeness. |
|  | edge_density | Calculated as the ratio of actual edges to possible edges (n*(n-1)/2 for undirected graphs). |
|  | assortativity_degree | Calculated as the Pearson correlation coefficient between the degrees of connected nodes. |
|  | transitivity | Calculated as the ratio of triangles to connected triples in the network. |
|  | cluster_number | The number of clusters typically calculated using algorithms like Louvain, Infomap, or walktrap. |
|  | modularity | Calculated based on the difference between actual edges within communities and expected edges in a random network. |
|  | node_number | Simply the count of all nodes (vertices) in the network. |
|  | edge_number | Simply the count of all edges in the network. |
|  | diameter | The longest shortest path between any two nodes in the network. |
|  | Percent postive edges (PPE) | Calculated as the ratio of positive edges to total edges in signed networks. |
|  | infocentrality | Calculated using the information of all paths between nodes, considering both direct and indirect connections. |
|  | efficiency | Calculated as the average of the inverse of the shortest path lengths between all node pairs. |
|  | connectance | Similar to edge density but often used in ecological networks. |
|  | connectivity_entropy | Calculated using Shannon entropy on the degree distribution of the network. |

**Table** **S3**. The results of pairwise permutation-based t tests (perm-Ttest) for seawater environmental properties. The analyses were done for comparing different regions in each layer, and for comparing different layers in each region, respectively. The significance values (*P*) were adjusted with the ‘fdr’ method for multiple comparisons. Values less than 0.05 (*P* < 0.05) were shown in bold text. TOC, total organic carbon; Temp., temperature; Sal., salinity; Dep., depth.

|  | pairwise comparisons | **NO_3_^-^** | **PO_4_^3-^** | **TOC** | **NH_4_^+^** | **Temp.** | **Sal.** | **pH** | **Dep.** |
| --- | --- | --- | --- | --- | --- | --- | --- | --- | --- |
| *In each layer* |  |  |  |  |  |  |  |  |  |
| Bottom | Hainan V.S. ZhongXisha | 0.77 | 0.61 | **0.006** | **0.015** | **0.003** | 0.19 | **0.012** | **0.006** |
|  | Hainan V.S. Nansha | 0.77 | **0.006** | **0.008** | **0.015** | **0.003** | 0.24 | **0.024** | **0.006** |
|  | ZhongXisha V.S. Nansha | 0.77 | **0.09** | **0.006** | 0.16 | **0.01** | 0.31 | 0.276 | 0.444 |
| Surface | Hainan V.S. ZhongXisha | 0.51 | 0.28 | **0.008** | 0.252 | **0.003** | 0.096 | 0.14 | 0.070 |
|  | Hainan V.S. Nansha | 0.41 | 0.15 | **0.006** | 0.072 | **0.003** | 0.297 | 0.14 | **0.024** |
|  | ZhongXisha V.S. Nansha | 0.41 | 0.28 | **0.008** | 0.054 | **0.008** | 0.722 | 0.3 | 0.051 |
| *In each region* |  |  |  |  |  |  |  |  |  |
| Hainan | Bottom V.S. Surface | 0.43 | 0.45 | 0.2 | 0.85 | 0.98 | 0.51 | 0.55 | **0.002** |
| ZhongXisha | Bottom V.S. Surface | 0.88 | 0.57 | 0.42 | 0.13 | **0.014** | 0.44 | 0.45 | **0.002** |
| Nansha | Bottom V.S. Surface | 0.18 | 0.2 | 0.28 | 0.95 | 0.084 | 0.54 | 0.96 | **0.014** |

**Table S4.** The three-way permutation based ANOVA (perm-ANOVA) tests on alpha diversity. The *P* values less than 0.05 were shown in bold. For simplicity, no interactions between factors were included in the models. Df, degree of freedom.

| *Alpha diversity* |  | **Df** | **Sum of square** | **Mean square** | **Iteration** | ***P*** |
| --- | --- | --- | --- | --- | --- | --- |
| Richness | Region | 2 | 1058843 | 529422 | 5000 | **<2e-16** |
|  | Lifestyle | 1 | 69430 | 69430 | 906 | 0.10044 |
|  | Layer | 1 | 52548 | 52548 | 1105 | 0.08326 |
|  | Residuals | 78 | 1880408 | 24108 |  |  |
| Pielou’s Evenness | Region | 2 | 0.0253 | 0.0126 | 5000 | **<2e-16** |
|  | Lifestyle | 1 | 0.000003 | 0.000003 | 51 | 0.9216 |
|  | Layer | 1 | 0.000185 | 0.000185 | 162 | 0.3827 |
|  | Residuals | 78 | 0.022090 | 0.000283 |  |  |

**Table** **S5**. The results of pairwise permutation-based t (perm-Ttest) tests for alpha diversity (richness and Pielou’s evenness). The analyses were done for comparing different regions in each layer, for comparing different layers in each region, and for comparing PA and FL communities in each region and layer, respectively. The significance values (*P*) were adjusted with the ‘fdr’ method for multiple comparisons. Values less than 0.05 (*P* < 0.05) were shown in bold text.

|  | **Pairwise comparisons** | **FL communities** | |  | **PA communities** | |
| --- | --- | --- | --- | --- | --- | --- |
|  |  | **Richness** | **Pielou’s evenness** |  | **Richness** | **Pielou’s evenness** |
| *In each layer* |  |  |  |  |  |  |
| Bottom | Hainan V.S. ZhongXisha | **0.006** | **0.003** |  | **0.024** | **0.018** |
|  | Hainan V.S. Nansha | **0.024** | **0.003** |  | 0.562 | 0.46 |
|  | ZhongXisha V.S. Nansha | 0.070 | 0.128 |  | 0.111 | **0.021** |
| Surface | Hainan V.S. ZhongXisha | **0.003** | **0.006** |  | **0.012** | **0.006** |
|  | Hainan V.S. Nansha | **0.003** | **0.027** |  | 0.296 | 0.364 |
|  | ZhongXisha V.S. Nansha | 1.000 | 0.31 |  | **0.045** | **0.012** |
| *In each region* |  |  |  |  |  |  |
| Hainan | Bottom V.S. Surface | 0.92 | 0.52 |  | 0.13 | 0.29 |
| ZhongXisha | Bottom V.S. Surface | 0.16 | 0.37 |  | 0.39 | 0.48 |
| Nansha | Bottom V.S. Surface | 0.62 | 0.93 |  | 0.42 | 0.44 |
|  |  |  | |  |  | |
| *In each region and layer* |  | **Richness** | |  | **Pielou’s evenness** | |
| Hainan-Bottom | PA V.S. FL | 0.370 | |  | 0.460 | |
| Hainan-Surface | PA V.S. FL | 0.500 | |  | 0.760 | |
| ZhongXisha-Bottom | PA V.S. FL | 0.240 | |  | 0.730 | |
| ZhongXisha-Surface | PA V.S. FL | 0.560 | |  | **0.012** | |
| Nansha-Bottom | PA V.S. FL | 0.076 | |  | 0.062 | |
| Nansha-Surface | PA V.S. FL | 0.086 | |  | 0.390 | |

**Table** **S6**. The results of pairwise permutation-based t tests (perm-Ttest) for the relative abundances of main phyla. The analyses were done for comparing different regions in each lifestyle, and for comparing different lifestyles in each region, respectively. The significance values (*P*) were adjusted with the ‘fdr’ method for multiple comparisons. Values less than 0.05 (*P* < 0.05) were shown in bold text.

|  | **Pairwise comparisons** | Gamaproteobacteria | Alphaproteobacteria | Bacteroidota | Cyanobacteria | Actinobacteriota | Planctomycetota | Verrucomicrobiota | Bdellovibrionota | Desulfobacterota | Marinimicrobia | Proteobacteia_unclassified | Myxococcota | Firmicutes | SAR324_clade |
| --- | --- | --- | --- | --- | --- | --- | --- | --- | --- | --- | --- | --- | --- | --- | --- |
| *In each lifestyle* |  |  |  |  |  |  |  |  |  |  |  |  |  |  |  |
| PA | Hainan V.S. ZhongXisha | **0.003** | **0.003** | **0.003** | **0.006** | **0.002** | **0.006** | **0.003** | 0.62 | 0.92 | **0.002** | **0.027** | 0.82 | **0.006** | **0.006** |
|  | Hainan V.S. Nansha | **0.003** | **0.003** | **0.003** | 0.100 | **0.002** | **0.006** | **0.003** | 0.84 | 0.048 | **0.002** | **0.006** | **0.048** | **0.018** | 0.318 |
|  | ZhongXisha V.S. Nansha | 0.092 | 0.926 | 0.364 | **0.015** | **0.002** | **0.026** | 0.274 | 0.62 | **0.006** | **0.002** | 0.258 | **0.006** | 0.548 | **0.006** |
| FL | Hainan V.S. ZhongXisha | **0.003** | **0.018** | **0.003** | 0.228 | **0.002** | **0.003** | **0.003** | 0.532 | 0.48 | **0.003** | 0.134 | 0.34 | **0.027** | **0.003** |
|  | Hainan V.S. Nansha | **0.003** | **0.006** | **0.003** | **0.012** | **0.002** | 0.82 | **0.008** | **0.003** | 0.48 | **0.048** | **0.003** | 0.93 | 0.672 | **0.04** |
|  | ZhongXisha V.S. Nansha | 0.620 | 0.48 | 0.556 | **0.006** | **0.002** | **0.003** | **0.003** | **0.003** | 0.54 | **0.003** | **0.003** | 0.52 | **0.006** | **0.003** |
| *In each region* |  |  |  |  |  |  |  |  |  |  |  |  |  |  |  |
| Hainan | PA V.S. FL | 0.45 | 0.13 | **0.002** | **0.002** | 0.66 | 0.35 | **0.012** | **0.04** | 0.19 | **0.002** | 0.61 | 0.68 | 0.21 | **0.004** |
| ZhongXisha | PA V.S. FL | **0.026** | **0.014** | 0.85 | **0.002** | **0.01** | **0.002** | 0.83 | **0.04** | 0.14 | 0.056 | 0.2 | **0.028** | **0.002** | **0.012** |
| Nansha | PA V.S. FL | 0.92 | 0.25 | **0.024** | **0.012** | **0.02** | **0.002** | **0.002** | 0.87 | **0.004** | **0.006** | 0.11 | **0.004** | **0.002** | 0.92 |

**Table S7**. The partial mantel analyses results for PA and FL community compositional changes. Values less than 0.05 (*P* < 0.05) were shown in bold text.

|  | PA | |  | FL | |
| --- | --- | --- | --- | --- | --- |
|  | r | *P* |  | r | *P* |
| NO_3_^-^ | -0.034 | 0.617 |  | 0.095 | 0.173 |
| PO_4_^3-^ | -0.151 | 1 |  | -0.097 | 0.894 |
| TOC | 0.362 | **0.001** |  | 0.408 | **0.001** |
| NH_4_^+^ | 0.184 | **0.008** |  | 0.25 | **0.006** |
| Temperature | 0.791 | **0.001** |  | 0.808 | **0.001** |
| Salinity | 0.278 | **0.002** |  | 0.254 | **0.002** |
| pH | 0.288 | **0.001** |  | 0.197 | **0.009** |
| Depth | 0.015 | 0.326 |  | -0.032 | 0.652 |
| PCNM1 | 0.116 | **0.044** |  | -0.058 | 0.821 |
| PCNM2 | 0.165 | **0.002** |  | 0.294 | **0.006** |

**Table** **S8**. The results of pairwise permutation-based t (perm-Ttest) tests for network traits (node-level). The analyses were done for comparing different regions in each lifestyle, and for comparing PA and FL communities in each region, respectively. The significance values (*P*) were adjusted with the ‘fdr’ method for multiple comparisons. Values less than 0.05 (*P* < 0.05) were shown in bold text. PPE, proportion of the positive edges; Betw., betweenness; Knn, the number of nodes linked with all the neighbors of one specific node; EigenC, eigen_centrality. The description about these traits can be found in the Table S2.

|  | **Pairwise comparisons** | **PPE** | **Betw.** | **Knn** | **Closeness** | **EigenC** | **Degree** |
| --- | --- | --- | --- | --- | --- | --- | --- |
| *In each lifestyle* |  |  |  |  |  |  |  |
| PA | Hainan V.S. ZhongXisha | 1.000 | 0.50 | **0.003** | 0.53 | 0.84 | 0.021 |
|  | Hainan V.S. Nansha | 1.000 | 0.50 | **0.003** | 0.53 | 0.84 | 0.021 |
|  | ZhongXisha V.S. Nansha | 1.000 | 0.88 | 0.512 | 0.59 | 0.84 | 0.694 |
| FL | Hainan V.S. ZhongXisha | **0.030** | 0.98 | 0.89 | 0.95 | 0.84 | 0.34 |
|  | Hainan V.S. Nansha | **0.024** | 0.98 | 0.89 | 0.95 | 0.84 | 0.34 |
|  | ZhongXisha V.S. Nansha | 0.862 | 0.98 | 0.89 | 0.95 | 0.97 | 0.95 |
| *In each region* |  |  |  |  |  |  |  |
| Hainan | PA V.S. FL | 0.75 | 0.49 | **0.002** | **0.002** | **0.014** | **0.002** |
| ZhongXisha | PA V.S. FL | **0.008** | 0.91 | 0.13 | **0.002** | 0.052 | 0.84 |
| Nansha | PA V.S. FL | **0.006** | 0.99 | **0.032** | **0.002** | **0.014** | 0.51 |

**Table** **S9**. The results of pairwise permutation-based t (perm-Ttest) tests for network robustness. The analyses were done for comparing different regions in each lifestyle, and for comparing PA and FL communities in each region, respectively. The significance values (*P*) were adjusted with the ‘fdr’ method for multiple comparisons. Values less than 0.05 (*P* < 0.05) were shown in bold text. robustness_rdm, robustness calculated by random removing nodes; robustness_tgt, robustness calculated by removing nodes with top degrees (targeted).

|  | **Pairwise comparisons** | **robustness_rdm** | **robustness_tgt** |
| --- | --- | --- | --- |
| *In each lifestyle* |  |  |  |
| PA | Hainan V.S. ZhongXisha | **0.006** | **0.002** |
|  | Hainan V.S. Nansha | **0.018** | **0.002** |
|  | ZhongXisha V.S. Nansha | 0.178 | **0.002** |
| FL | Hainan V.S. ZhongXisha | **0.002** | **0.002** |
|  | Hainan V.S. Nansha | **0.002** | **0.002** |
|  | ZhongXisha V.S. Nansha | **0.002** | **0.002** |
| *In each region* |  |  |  |
| Hainan | PA V.S. FL | **0.002** | **0.002** |
| ZhongXisha | PA V.S. FL | **0.002** | **0.002** |
| Nansha | PA V.S. FL | **0.002** | **0.002** |

**Table S10**. The top five bins contributing to the process of homogeneous selection in Hainan PA community. BinRCtb, the relative contribution of that bin to the process. BinRA, the relative abundance of all sequences from that bin in the whole community. RAinBin, The relative abundance of the top ASV in its bin. Top ASVs, the most abundant ASV in each bin. The Class, Family and Genus information for the top ASV was displayed. The number behind the taxa in parentheses denotes the relative abundance of that taxa in the bin.

| **Bin** | **BinRCtb** | **BinRA** | **Top ASV** | **RAinBin** | **Class** | **Family** | **Genus** |
| --- | --- | --- | --- | --- | --- | --- | --- |
| bin152 | 0.252 | 0.052 | ASV74 | 0.251 | Alphaproteobacteria (99.8) | Clade_I (99.7) | Clade_Ia (77.4) |
| bin248 | 0.121 | 0.026 | ASV278 | 0.399 | Acidimicrobiia (99.6) | Actinomarinaceae (99.6) | Candidatus_Actinomarina (99.6) |
| bin151 | 0.099 | 0.032 | ASV10 | 0.536 | Alphaproteobacteria (100) | Clade_I (100) | Clade_Ia (99.5) |
| bin260 | 0.064 | 0.023 | ASV65 | 0.325 | Bacteroidia (100) | Cryomorphaceae (100) | uncultured (99.6) |
| bin116 | 0.038 | 0.010 | ASV189 | 0.424 | Alphaproteobacteria (100) | Sphingomonadaceae (90.1) | Erythrobacter (83.4) |

**Figure S1.** The Venn plots showing the overlap of ASV ids between PA and FL communities in each region.

**Figure S2.** The Spearman correlations between water environmental properties and the relative abundances of main phyla for (A) FL and (B) PA communities. TOC, total organic carbon; Temp, temperature; Sal, salinity; Dep, depth. The significant (FDR-adjusted *P* < 0.05) correlation coefficients were shown in the cells.

**Figure S3.** The relative importance of different ecological processes in driving the assembly of bacterial communities. FL, free-living; PA, particle-attached. The process abbreviations, HeS: heterogeneous selection; HoS: homogeneous selection; DL: dispersal limitation; HoD: homogeneizing dispersal; Drift: stochastic drift.

**Figure S4.** The node-level network topological traits for community networks from different regions and lifestyles. PPE, percent of positive edges; Knn, the sum of degree of the neighbors of one specific node. The descriptions for the network traits can be found in Table S2. FL, free-living; PA, particle-attached. Sharing no common letters denotes significant differences (permTtest, FDR-adjusted *P* < 0.05) of the mean values between different regions for FL and PA communities, respectively. The “**” and “*” denote significant differences of the mean values between different lifestyles for a specific region (permTtest, *P* < 0.01 and *P* < 0.05, respectively).

**Figure S5.** The *Zi-Pi* plots for FL networks from (A) Hainan, (B) ZhongXisha and (C) Nansha. J-L, the *Zi-Pi* plots for PA networks from (D) Hainan, (E) ZhongXisha and (F) Nansha. The taxa information (phylum/subphylum) of the keystone species was shown in text and the text was colored based on the taxa group. Some texts’ positions were jittered for a better illustration. The definition and calculation of keystone species are referred from Guimera and Amaral (1), and Olesen et al. (2).

**
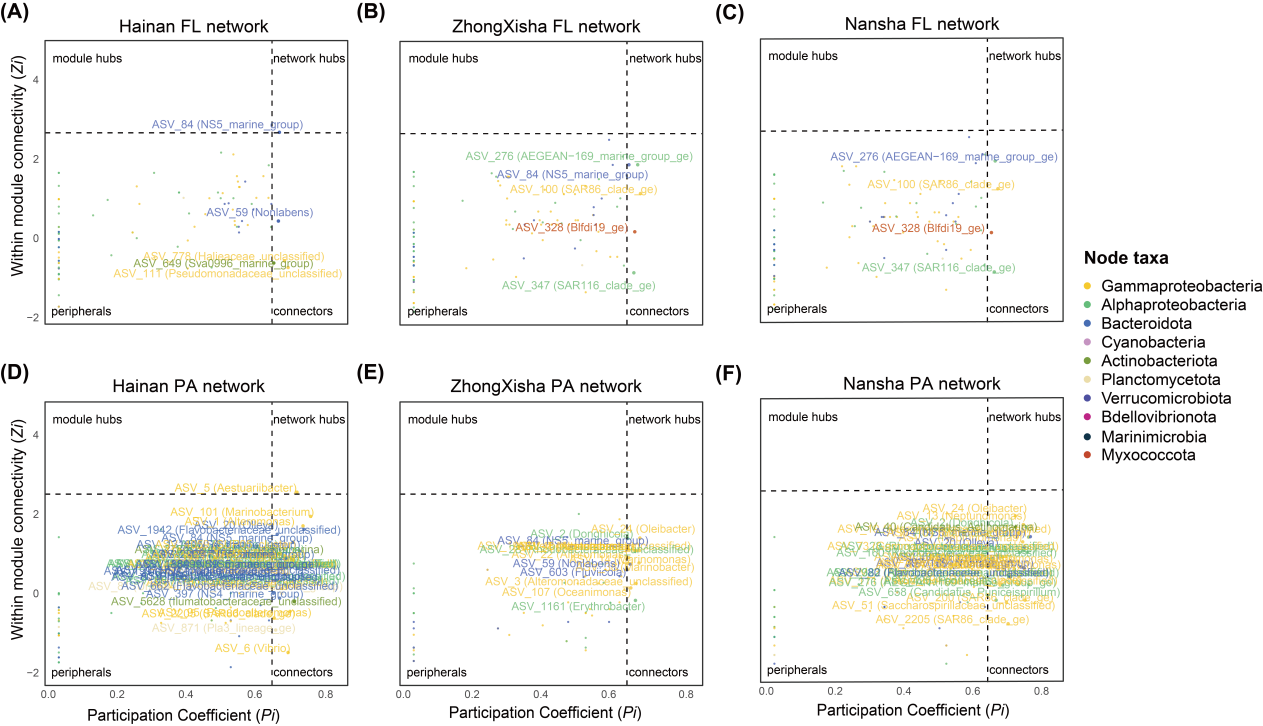
**

**Figure S6**. The mantel correlation relationships between water properties with the relative abundances of the keystone species and network-level topological traits (data table comprised of the traits introduced in the Table S2) for (A) free-living (FL) community networks and (B) particle-attached (PA) community networks. Temp, temperature; Sal, salinity; Dep, depth.

**Figure S7.** The correlation between the relative abundances of the main genera from the top 5 bins contributing to the selection process for Hainan PA community assembly with the seawater environmental properties. TOC, total organic carbon. The correlation coefficients with *P* values more than 0.05 (not significant) were added a “×” symbol above.

**Reference**

1. Guimera R, Amaral LAN. 2005. Functional cartography of complex metabolic networks. Nature 433:895e900. https://doi.org/10.1038/nature03288

2. Olesen JM, Bascompte J, Dupont YL, Jordano P. 2007. The modularity of pollination networks. Proc Nat Acad Sci USA 104:19891e19896. https://doi.org/10.1073/pnas.070637510
